# Impaired Cognitive Flexibility After Neonatal Perirhinal Lesions in Rhesus Macaques

**DOI:** 10.3389/fnsys.2019.00006

**Published:** 2019-01-30

**Authors:** Alison R. Weiss, Jessica White, Rebecca Richardson, Jocelyne Bachevalier

**Affiliations:** ^1^Department of Psychology, Emory University, Atlanta, GA, United States; ^2^Oregon National Primate Research Center, Beaverton, OR, United States; ^3^Yerkes National Primate Research Center, Atlanta, GA, United States

**Keywords:** excitotoxic lesion, attentional set-shifting, reversal learning, perseveration, proactive interference

## Abstract

Previous research indicated that monkeys with neonatal perirhinal lesions (Neo-PRh) were impaired on working memory (WM) tasks that generated proactive interference, but performed normally on WM tasks devoid of interference (Weiss et al., 2016). This finding suggested that the early lesions disrupted cognitive processes important for resolving proactive interference, such as behavioral inhibition and cognitive flexibility. To distinguish between these possibilities, the same Neo-PRh monkeys and their controls were tested using the Intradimensional/Extradimensional attentional set-shifting task (Roberts et al., 1988; Dias et al., 1997). Neo-PRh monkeys completed the Simple and Compound Discrimination stages, the Intradimensional Shift stage, and all Reversal stages comparably to controls, but made significantly more errors on the Extradimensional Shift stage of the task. These data indicate that impaired cognitive flexibility was the likely source of increased perseverative errors made by Neo-PRh monkeys when performing WM tasks, rather than impaired behavioral inhibition, and imply that the perirhinal cortex and its interactions with the PFC may play a unique and critical role in the development of attentional set shifting abilities.

## Introduction

A recent study reported that adult monkeys with neonatal lesions of the perirhinal cortex (Neo-PRh) had working memory (WM) impairments that were characterized by a tendency to make perseverative errors on tasks that generated proactive interference (Weiss et al., 2016). However, the same Neo-PRh animals were unimpaired when tested with a WM task that was devoid of interference (Weiss et al., 2016). Taken together, these data suggested that the Neo-PRh lesions may have resulted in difficulty resolving proactive interference, rather than a deficit in WM *per se*.

Resolving proactive interference requires suppressing behavioral responses based on “old” information, and flexibility to shift cognitive resources toward learning/remembering “new” information (Jha et al., 2004). Therefore, the increased perseverative errors made by the Neo-PRh monkeys could be due to either a failure to suppress the influence of previously acquired stimulus-reward associations (i.e., behavioral inhibition), resulting in repetitive tendencies, or to difficulty shifting attention toward new stimulus-reward associations (i.e., cognitive flexibility), resulting in a tendency to choose the previously rewarded stimulus.

Lesion studies in monkeys have already demonstrated a double-dissociation between behavioral inhibition supported by the orbitofrontal cortex (OFC), and cognitive flexibility supported by the ventrolateral prefrontal cortex (vlPFC) (Dias et al., 1997; Rogers et al., 2000; Monchi et al., 2001; Burnham et al., 2010; Bissonette et al., 2013). Given that the PRh has robust interconnections with both of these cortical areas (Barbas and Pandya, 1989; Suzuki and Amaral, 1994a,b; Lavenex et al., 2002; Petrides and Pandya, 2002), it is possible that the PRh also contributes in mechanisms underlying behavioral inhibition and/or cognitive flexibility. Therefore, the goal of this study was to distinguish between these possible alternatives by characterizing the ability of the same Neo-PRh monkeys to perform a task that taps both capacities, i.e., the Intradimensional-Extradimensional set-shifting paradigm (ID-ED) (Roberts et al., 1988).

## Materials and Methods

The Institutional Animal Care and Use Committee (IACUC) at Emory University in Atlanta, GA, United States, approved all experimental protocols. All guidelines specified in the NIH Guide for the Care and Use of Laboratory Animals (National Research Council (US), 2011) were strictly followed.

### Subjects

Eleven adult rhesus macaques, aged 9–16 years, participated in this experiment (7 females, 4 males). Between 7 and 12 days postnatal, 6 monkeys received bilateral injections of ibotenic acid to the perirhinal cortex (group Neo-PRh; 3 females, 3 males), and 2 received sham surgeries (group Neo-C; 1 female, 1 male). One animal did not undergo any surgical or anesthetic procedures (Neo-UC; 1 female). All of these subjects received the same rearing conditions, which included extensive socialization opportunities with age-matched peers and human caregivers (for detailed description of rearing procedures see Goursaud and Bachevalier, 2007; Raper et al., 2013). All monkeys, except Neo-C-1, were born at the Yerkes National Primate Research Center (Lawrenceville, GA, United States). Neo-C-1 was born at the University of Texas M.D. Anderson Cancer Center Science Park (Bastrop, TX, United States), and moved to the Yerkes NPRC.

Two additional monkeys received sham operations in adulthood (Adult-C; 1 female; 1 male) and were available to participate in behavioral testing. These Adult-C animals were mother-raised in a large colony of macaques at the Yerkes NPRC Field Station under a semi-naturalistic environment (see Raper et al., 2013 for more details), and moved to indoor pair housing between 2 and 4 years of age.

At the time of this experiment, all monkeys were housed individually in rooms with 12-h light/dark cycles (7 AM/7 PM), fed Purina Old World Primate chow (formula 5047) and supplemented with fresh fruit enrichment. During testing, the food ration was given once daily following testing, and adjusted individually to ensure that the animals were motivated to perform on the task and maintained their weight at 85% or above of their free-feeding weight. Water was given *ad libitum*.

### Neuroimaging and Surgical Procedures

Between 10 and 12 days of age, subjects in groups Neo-PRh and Neo-C underwent surgery to create excitotoxic lesions of the perirhinal cortex using ibotenic acid, or sham operations, respectively. Animals in the Adult-C group were between 6 and 12 years of age at the time of their sham surgeries.

The brain was imaged with a 3T Siemens Magnetom Trio system (Siemens Medical Solutions, Malvern, PA, United States at YNPRC) using a 5 cm surface coil. Both pre-surgery and 1 week post-surgery, two sets of images were obtained: (1) high-resolution structural T1 images [3D T1-weighted fast spoiled gradient (FSPGR)-echo sequence, TE = 2.6 ms, TR = 10.2 ms, 25° flip angle, contiguous 1 mm sections, 12 cm FOV, 256 × 256 matrix]; and (2) Fluid Attenuated Inversion Recovery (FLAIR) images, [TE = 140 ms, TR = 1000 ms, inversion time (TI) = 2200 ms, contiguous 3 mm sections, 12 cm FOV, 256 × 256 matrix; image sequences acquired in three series offset 1 mm posterior]. The T1-weighed images were used to calculate the injection sites pre-surgery and the FLAIR images were used to estimate the extent of PRh damage as well as damage to adjacent structures, as described in the section below.

Throughout the duration of the pre-surgical MRI scans, subjects were sedated (10 mg/kg of 7:3 Ketamine Hydrochloride, 100 mg/ml, and Xylazine, 20 mg/ml, administered i.m.) and intubated to allow inhalation of isoflurane (1–2%, v/v) and maintain in an appropriate plane of anesthesia. The subject’s head was restrained in a stereotaxic apparatus and an IV drip (0.45% NaCl and dextrose) was used to maintain normal hydration. Vital signs (heart and respiration rates, blood pressure, body temperature and expired CO_2_) were constantly monitored during the scan and surgical procedures that followed.

Following the pre-surgical scans, animals were immediately transported to the operating room and maintained under deep anesthesia with Isoflurane gas (1–2%, v/v, to effect) throughout the surgical procedures, which were performed using aseptic conditions. The scalp was shaved and cleaned with chlorhexidine diacetate (Nolvasan, Pfizer). Bupivacaine Hydrochloride (Marcaine 25%, 1.5 ml), a long-lasting local anesthetic, was injected along the planned midline incision of the scalp, which extended from the occipital to the orbital ridges. Bilateral craniotomies (1 cm wide × 2.5 cm long) were made above the areas to be injected. The Neo-PRh group was given injections 2 mm apart along the rostral-caudal length of the perirhinal cortex using 0.4 μl ibotenic acid (Biosearch Technologies, Novato, CA, United States 10 mg/ml in PBS, pH 7.4, at a rate of 0.2 μl/min).

Animals in the Neo-C and Adult-C groups underwent the same procedures, except that the injection needles were not lowered into the brain. At completion of surgery, the dura, galea, and skin were closed in anatomical layers and the animals removed from isoflurane, extubated, and closely monitored until complete recovery from anesthesia. Analgesic (acetaminophen, 10 mg/kg PO) was given QID for 3 days after surgery. Additionally, animals received dexamethazone sodium phosphate (0.4 mg/kg IM) to reduce edema and Cephazolin (25 mg/kg IM) SID starting 12 h prior to surgery and ending 7 days after to prevent infection.

### Lesion Assessment

Lesion extent was estimated using MRI images (coronal FLAIR) acquired 1-week post-surgery. In this post-surgical scan, edema caused by cell death after the excitotoxin injections is visible as hypersignals. Lesion extent was evaluated with methods described in detail by Zeamer et al. (2015) and briefly summarized here. After identifying the areas of hypersignals on each MR image through the perirhinal cortex, the extent of hypersignals were plotted onto matching coronal sections of a normal monkey brain. The surface area (in pixels^2^) of damage to the left and right perirhinal cortex and any unintended damage to adjacent structures was then measured using Image J^®^ software. Calculations of the percentage of volume of damage were done by dividing the volume of damage to the perirhinal cortex by the volume of the perirhinal cortex in a normal monkey of the same age and multipling by 100. A similar procedure was used to calculate additional damage to adjacent structures (e.g., hippocampus, amygdala, and entorhinal cortex).

**FIGURE 1:**
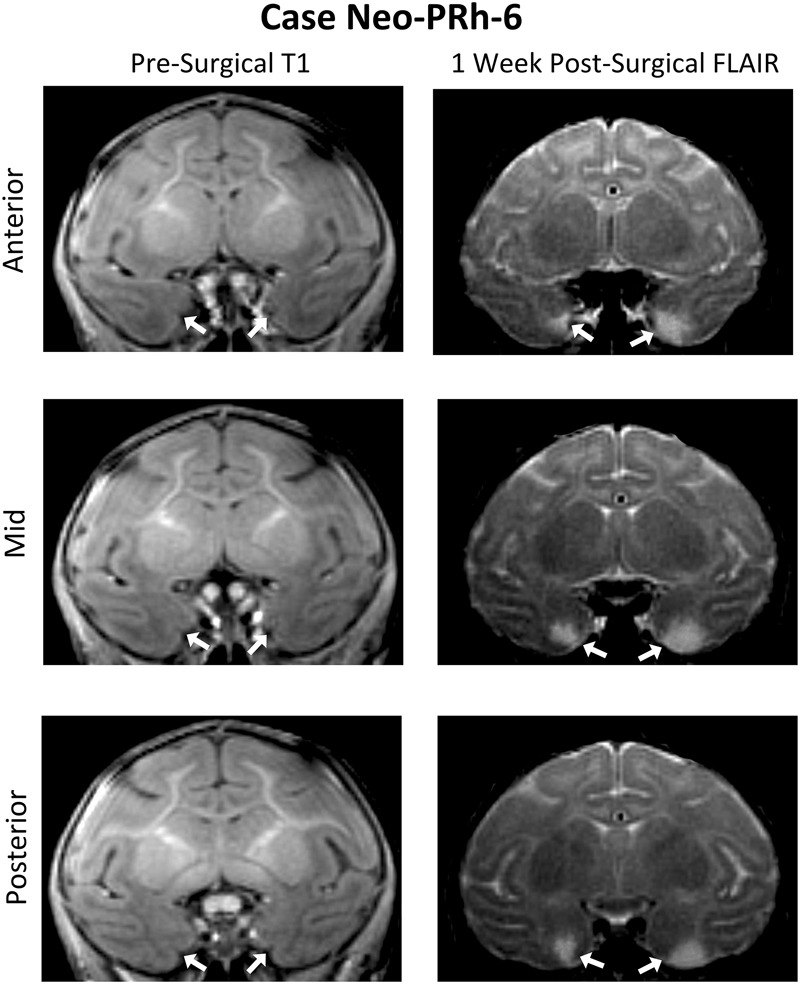
Pre- and post-Surgical MR Images from a representative case (Neo-PRh-6). MR images shown at three rostro-caudal levels through the perirhinal cortex are pre-surgical structural T1 weighted coronal images (left column) and 1 week post-surgical coronal FLAIR images (right column) for a representative case. Visible in the post-surgical images are regions of hypersignal (white areas) that are indicative of edema and cell damage resulting from the ibotenic acid injection. Arrows point to the rhinal sulcus (left column) and to areas of hypersignal (right column). See Zeamer et al. (2015), Weiss and Bachevalier (2016), Weiss et al. (2016, 2017), and Ahlgrim et al. (2017) for illustration of lesion extent for additional Neo-PRh cases.

Table 1 summarizes the extent of intended and unintended damage for each surgical case. The ibotenic acid injections resulted in extensive bilateral PRh damage in all cases (average = 73.60%, min = 67.06%, and max = 83.34%). The injections also caused mild unintended entorhinal damage (average = 20.57%, min = 5.42%, and max = 34.49%). Figure 1 shows pre-surgical and post-surgical MR images of a representative case (Neo-PRh-6). Images from additional cases have been previously published (Zeamer et al., 2015; Weiss and Bachevalier, 2016; Weiss et al., 2016, 2017; Ahlgrim et al., 2017).

**Table 1:** Summary of lesion extent.

Subjects	PRh				ERh				TE			
	L%	R%	X%	W%	L%	R%	X%	W%	L%	R%	X%	W%
Neo-PRh-1	89.76	79.91	83.34	69.04	28.51	2.28	15.39	0.65	4.53	9.70	7.11	0.44
Neo-PRh-2	68.16	70.58	69.37	48.11	17.72	20.65	19.19	3.36	0.14	0.06	0.10	0.00
Neo-PRh-3	65.45	81.02	73.23	53.02	7.72	3.12	5.42	0.24	0.26	3.39	1.82	0.01
Neo-PRh-4	59.40	74.73	67.06	44.39	11.55	17.84	14.69	2.06	0.72	2.62	1.67	0.02
Neo-PRh-5	75.90	66.81	71.35	50.71	38.60	29.86	34.32	11.53	0.72	0.41	0.57	0.00
Neo-PRh-6	74.12	80.31	77.22	59.53	25.34	43.64	34.49	11.06	0.37	2.93	1.65	0.01
*Average*	*72.13*	*75.06*	*73.60*	*54.13*	*21.57*	*19.57*	*20.57*	*4.87*	*1.12*	*3.19*	*2.15*	*0.08*

**Subjects**	**TH/TF**				**AMY**				**HF**			
	**L%**	**R%**	**X%**	**W%**	**L%**	**R%**	**X%**	**W%**	**L%**	**R%**	**X%**	**W%**

Neo-PRh-1	0.00	0.00	0.00	0.00	8.24	10.86	9.55	0.89	0.13	2.39	1.26	0.00
Neo-PRh-2	0.00	0.00	0.00	0.00	0.00	2.76	1.38	0.00	0.00	0.00	0.00	0.00
Neo-PRh-3	0.00	0.00	0.00	0.00	0.00	0.00	0.00	0.00	0.00	0.27	0.14	0.00
Neo-PRh-4	0.00	0.00	0.00	0.00	0.00	0.00	0.00	0.00	0.00	0.00	0.00	0.00
Neo-PRh-5	7.02	3.93	5.47	0.28	0.00	0.00	0.00	0.00	3.37	0.00	1.68	0.00
Neo-PRh-6	0.00	0.00	0.00	0.00	3.78	4.17	3.97	0.16	3.32	0.32	1.77	0.01
*Average*	*1.17*	*0.66*	*0.91*	*0.05*	*2.00*	*2.96*	*2.48*	*0.18*	*1.12*	*0.50*	*0.81*	*0.00*

*Scores are estimates of intended and unintended damage following Neo-PRh lesions for each case. L% = percent damage to left hemisphere; R% = percent damage to right hemisphere; X% = average damage to both hemispheres; W% = weighted damage to both hemispheres [W% = (L% × R%)/100]. PRh, perirhinal cortex; ERh, entorhinal cortex, TE, temporal cortical area; TH/TF, parahippocampal cortex; AMY, amygdala; HF, hippocampal formation. Lesion extents from cases Neo-PRh-1 thru Neo-PRh-6 were previously reported by Zeamer et al. (2015).*

### Behavioral Testing

Prior to participating in this study, all subjects had experience with cognitive tests including concurrent discrimination learning, reinforcer devaluation, object reversal learning, safety signal learning, and emotional regulation (Ahlgrim et al., 2017). The Neo-PRh, Neo-C and Neo-UC monkeys had additional experience with tests of object recognition (Zeamer et al., 2015; Weiss and Bachevalier, 2016), WM (Weiss et al., 2016), perceptual discrimination and familiarity judgments (Weiss et al., 2017).

### Apparatus

The ID-ED task was conducted in a soundproof testing chamber with an automated testing apparatus. This apparatus consisted of a 3M Microtouch Touch Screen monitor and MedAssociates mini M&M dispenser controlled by a custom-written program using Presentation software. Before beginning the ID-ED task, monkeys were acclimated to the testing chamber, the touch screen, and the sound of the reward (M&M) dispenser in 15-min sessions for 3 consecutive days. After these sessions, the animals readily triggered the screen and ate the rewards as they were dispensed.

### Interdimensional-Extradimensional (ID-ED) Set-Shifting Task

**FIGURE 2:**
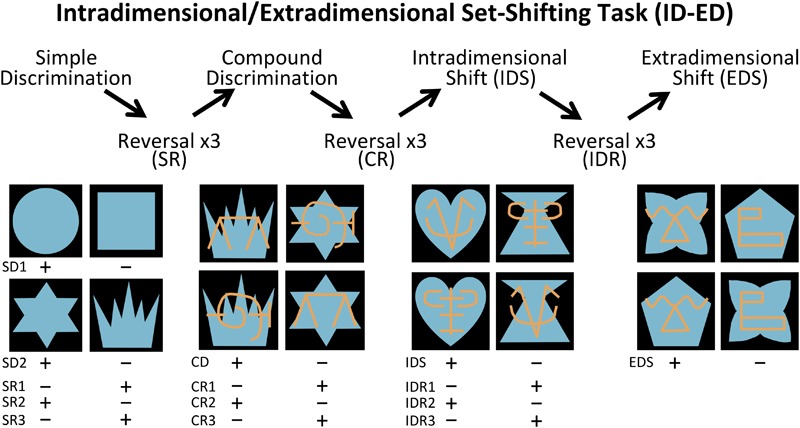
Intradimensional-extradimensional (ID/ED) Set Shifting Task Schematic. In the ID-ED paradigm, monkeys learned the series of discrimination problems and reversals illustrated here. They first learned two simple discrimination problems using blue shapes (SD1 and SD2). After reaching criterion on discrimination SD2, they received 3 successive reversals of SR1 to SR3. They were then given a compound discrimination CD in which the blue shapes of the last SR3 discrimination was overlayed with orange lines, but monkeys had to continue to respond to the blue shapes and ignore the orange lines. After reaching criterion on CD, they received 3 successive reversals of this discrimination problem (CR1 to CR3) following which they were moved to the Intradimensional shift (IDR), in which new blue shapes and orange lines were used but monkeys continued to respond to blue shapes. After three successive reversals of IDS (IDS1 to IDS3), they were moved to the Extradimensional Shit (EDS) in which new blue shapes and orange lines were used but this time the animals had to respond to the orange lines and ignore the blue shapes. Criterion was set at 10 correct choices in a row before moving to the next stage. Plus indicates stimulus rewarded and minus indicates unrewarded stimulus for each discrimination and reversal.

The Interdimensional-Extradimensional (ID-ED) set shifting task was based on the Wisconsin Card sort paradigm and closely resembled the version in the CANTAB battery of tasks (Roberts et al., 1988; Sahakian and Owen, 1992). For this study, a daily session consisted of 60 trials separated by 10 s inter-trial intervals. Each trial required a choice between two stimuli, one associated with a food reward (+) and the other not (-). The left-right positions of the rewarded and non-rewarded stimuli were varied pseudorandomly across 60 daily trials. Monkeys learned a series of discrimination problems (schematically illustrated in Figure 2 and described below), and advanced from one stage to the next after reaching the criterion of 10 correct trials in a row. If the monkey did not reach criterion within the daily testing session, they restarted the next day at the same stage but with their number of errors reset to zero. We recorded the number of errors to reach the learning criterion for each stage.

The first stage was a simple discrimination (SD1) between two blue shapes (S_1_+ and S_2_-). This stage was repeated a second time (SD2) using novel stimuli (S_3_+ and S_4_-) to ensure that the animals had fully acclimated to the testing chamber and were sufficiently motivated to complete 60 trials each session. SD2 was followed by a series of 3 reversals (SRs) using the same SD2 stimuli but with the reward contingency between S_3_+ and S_4_- switching for each reversal. Performance on reversals assessed behavioral inhibition by requiring subjects to learn to suppress responses toward a previously reinforced stimulus and to switch to a previously non-reinforced stimulus. Once monkeys completed three reversals, a second dimension was introduced to the stimuli; the blue shapes were overlaid with orange lines (L_A_ and L_B_). This third stage involved discrimination between compound (shape+line) stimuli (compound discrimination, CD). Importantly, on half the trials L_A_ overlay S_3_ and L_B_ overlay S_4_, and on the other half L_A_ overlay S_4_ and L_B_ overlay S_3_. Therefore, in the CD stage monkeys learned to respond selectively to the S+ shape regardless of which line (L_A_ or L_B_) was associated with the shape. When the monkeys learned this new discrimination, they completed another series of three reversals (compound reversals, CR). Following the CR stage, an Intradimensional Shift (IDS) was given, in which a new set of compound shape-line stimuli were introduced, and monkeys transferred the rule of responding to shape (S+) and ignoring the lines. Upon completing the IDS, there was another series of three reversals between the S+/S- (Intradimensional reversals, IDR). The final stage was an Extradimensional Shift (EDS) in which a new set of compound shape-line stimuli were introduced, but now monkeys were rewarded for choosing a specific line stimulus (L+) rather than the shape. Performance on the EDS stage assessed cognitive flexibility.

### Data Analysis

The errors of Adult-C and Neo-C groups were compared on all stages of the task using independent-sample *t*-tests. In no instances did the group differences reach significance. Additionally, the number of errors made by the Neo-UC animal fell within the standard deviations of group Neo-C and Adult-C for all stages, and so data from these three control groups were combined to form a single comparison group for all subsequent analyses (group Control).

To assess group differences in the ability to learn the reversal contingencies across stages, we compared the total number of errors to complete each series of reversals using a Group x Reversal type ANOVA with repeated measures for the second factor. Planned comparisons between groups for each reversal type were run using independent-sample *t*-tests, and between stages for the each group individually using paired-samples *t*-tests.

Similarly, to assess group differences in the ability to learn the simple (SD) and compound discrimination (CD) problems as well as the intradimensional (ID) and extradimensional (ED) discrimination problems, the numbers of errors across all discrimination problems were analyzed using a Group X Stage ANOVA with repeated measures for the second factor. Additional planned independent-sample *t*-tests were run between groups for each discrimination stage individually, and paired-sample *t*-tests between performances of the same group on different stages. Effect sizes were reported for all ANOVAs using partial eta squared (η_p_^2^). Effect sizes were reported for all *T*-tests using Cohen’s *d* (d_Cohen_).

All analyses were also run using sex as a second independent factor to determine whether there were any female/male differences among the groups. None of the analyses revealed significant sex effects, and so both sexes were combined for all analyses reported in the Results section.

Finally, bivariate Pearson correlations were run to determine if the extent of PRh damage, or unintended damage in the adjacent entorhinal cortex (ERh), was related to the number of errors to reach criterion at each stage of the ID/ED task.

## Results

**FIGURE 3:**
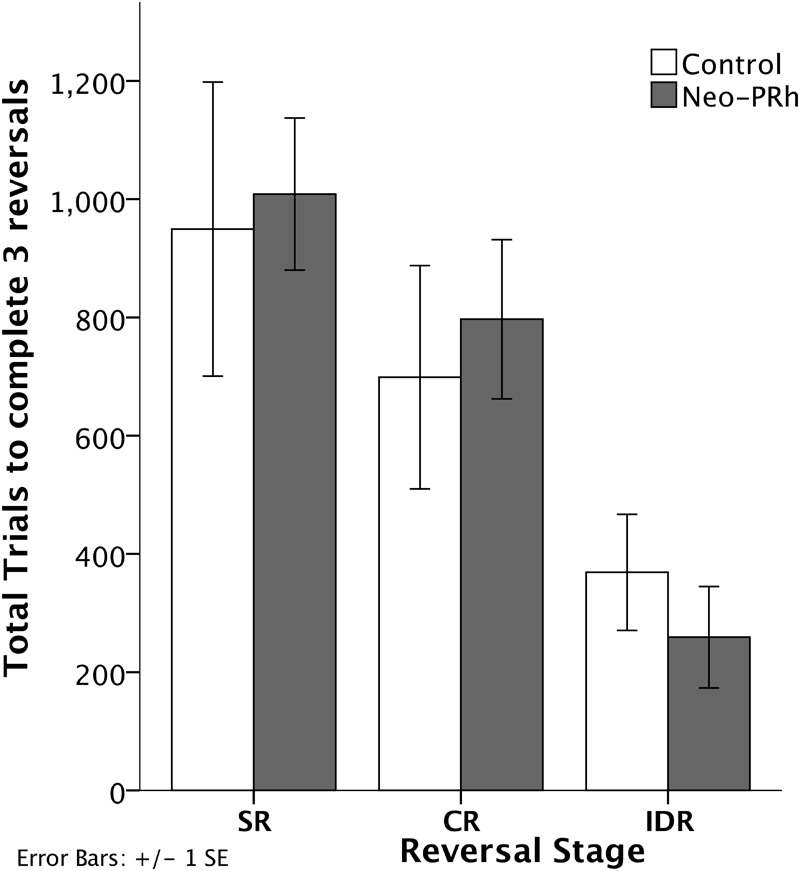
Reversal Stages. Total number of errors to complete the Simple Reversal (SR), Compound Reversal (CR), and Intradimensional Reversal (IDR) Stages for animals in the Neo-PRh group (shaded bars) and the Control group (open bars). Vertical lines represent ±1 SE.

The numbers of errors required to complete each reversal stage are illustrated in Figure 3. The Neo-PRh and Control groups made similar numbers of errors on average during each of the reversal stages, and both groups tended to make fewer errors as they advanced through the reversal stages. Table 2 reports the scores of the individual animals on each of the reversal stages. Analyses revealed a significant main effect of reversal stage [*F*(2,18) = 24.687, *p* = 0.001, η_p_^2^ = 0.733] but no significant main effect of group [*F*(1,9) = 0.01, *p* = 0.921, η_p_^2^ = 0.001] and no interaction [*F*(2,18) = 0.690, *p* = 0.514, η_p_^2^ = 0.071]. Planned independent-sample *t*-tests indicated that the groups did not differ significantly at any stage [SR: *t*(9) = -0.483, *p* = 0.640, d_Cohen_ = 0.293; CR: *t*(9) = -0.114, *p* = 0.912, d_Cohen_ = 0.069; IDR: *t*(9) = 0.953, *p* = 0.366, d_Cohen_ = 0.577]. However, planned paired-sample *t*-tests indicated that both groups made significantly less errors in the IDR stage than the CR stage [Neo-PRh: *t*(5) = 4.089, *p* = 0.009, d_Cohen_ = 1.763; Control: *t*(4) = 3.476, *p* = 0.025, d_Cohen_ = 1.133] and the SR stage [Neo-PRh: *t*(5) = 4.950, *p* = 0.004, d_Cohen_ = 2.49; Control: *t*(4) = 2.898, *p* = 0.044, d_Cohen_ = 1.62]. The number of errors made by group Neo-PRh also significantly differed between the SR and CR stages [*t*(5) = 3.905, *p* = 0.011, d_Cohen_ = 0.939], but this difference did not reach significance for group Neo-C [*t*(4) = 1.593, *p* = 0.186, d_Cohen_ = 0.597].

**Table 2:** Summary of intradimensional-extradimensional (ID-ED) task performance.

Groups	Discrimination stages					Reversal stages		
	SD1	SD2	CD	*IDS*	EDS	SR	CR	IDR
**Neo-PRh**								
Neo-PRh-1	2	68	56	26	304	634	549	284
Neo-PRh-2	74	64	24	19	274	591	296	181
Neo-PRh-3	7	25	24	15	127	501	432	75
Neo-PRh-4	80	64	20	1	29	509	267	29
Neo-PRh-5	26	18	53	43	144	283	137	51
Neo-PRh-6	21	189	34	11	325	1033	634	126
*Average*	*35.00*	*71.33*	*35.17*	*19.17*	*200.50*	*591.83*	*385.83*	*124.33*
**Control**								
Neo-C-1	7	19	14	3	30	170	77	54
Neo-C-7	243	14	25	10	76	365	391	263
Neo-C-9	133	2	22	12	80	542	602	300
Adult-C-3	15	27	27	14	83	887	526	214
Adult-C-4	22	93	105	9	103	618	265	84
*Average*	*84.00*	*31.00*	*38.60*	*9.60*	*74.40*	*516.40*	*372.20*	*183.00*

*Scores are number of errors to reach criterion (10 correct trials in a row). Discrimination Stages: SD1, first simple discrimination; SD2, second simple discrimination; CD, Compound Discrimination; IDS, Intradimensional Shift; EDS, Extradimensional Shift. Reversal Stages: SR, simple reversal; CR, Compound Reversal; IDR, Intradimensional Reversal. Figure 2 illustrates the intradimensional/extradimensional task.*

**FIGURE 4:**
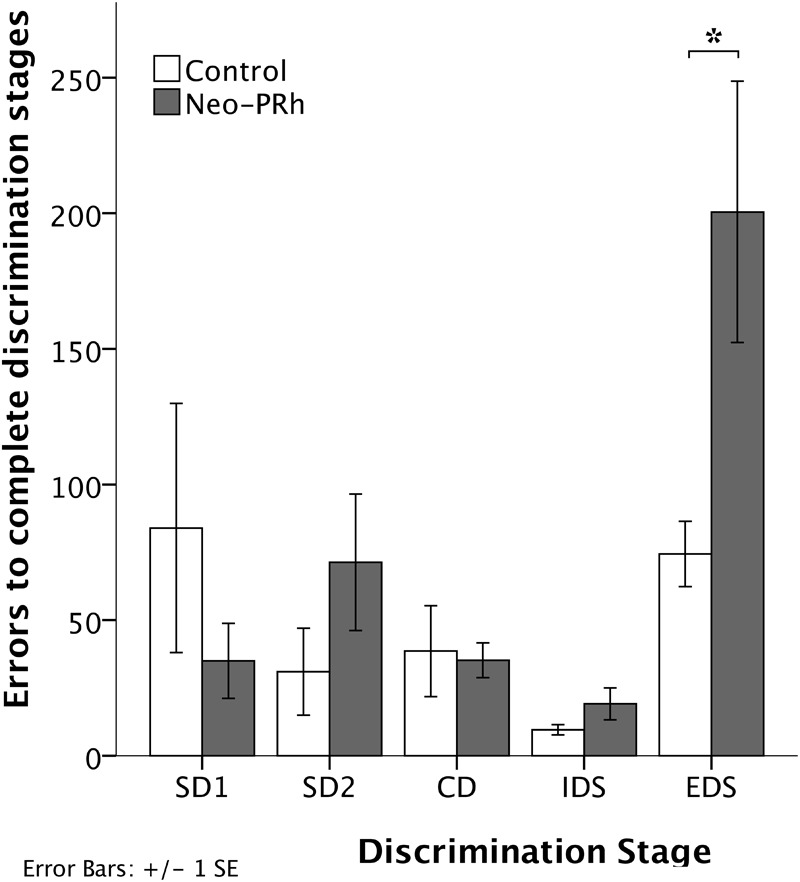
Discrimination Stages. Total number of errors to learn the Simple Discrimination (SD1 and SD2), Compound Discrimination (CD), Intradimensional Shift (IDS), and Extradimensional Shift (EDS) for animals in the Neo-PRh group (shaded bars) and the Control group (open bars). Vertical lines represent ±1 SE, and ^∗^indicates significant group differences (*p* < 0.05).

The numbers of errors that the Neo-PRh and Control groups required to complete each discrimination stage are illustrated in Figure 4 and summarized in Table 2. Both groups made similar numbers of errors on all of the discrimination stages, except the EDS stage where the Neo-PRh group made more than twice as many errors on average than the Control group (Neo-PRh = 200.5 errors; Control = 74.4 errors). Statistical analyses revealed no significant difference between the groups [*F*(1,9) = 2.032, *p* = 0.188, η_p_^2^ = 0.184], but the main effect of Stage and the Group X Stage interaction reached significance [*F*(4,36) = 7.385, *p* < 0.001, η_p_^2^ = 0.451 and *F*(4,36) = 3.606, *p* = 0.014, η_p_^2^ = 0.286, respectively]. Planned independent-sample *t*-tests revealed that the groups differed significantly on the EDS stage [*t*(9) = -2.320, *p* = 0.045, d_Cohen_ = 1.405] but not for any of the other stages [SD1: *t*(9) = 1.109, *p* = 0.296, d_Cohen_ = 0.672; SD2: *t*(9) = -1.287, *p* = 0.230, d_Cohen_ = 0.780; CD: *t*(9) = 0.206, *p* = 0.842, d_Cohen_ = 0.124, IDS: *t*(9) = -1.430, 0.186, d_Cohen_ = 0.866]. Additionally, paired sample *t*-tests indicated that both groups made more errors in the EDS stage than the IDS stage [Neo-PRh: *t*(5) = -3.833, *p* = 0.012, d_Cohen_ = 2.157; Control: *t*(4) = -6.028, *p* = 0.004, d_Cohen_ = 3.365], the CD stage [Neo-PRh: *t*(5) = -3.590, *p* = 0.016, d_Cohen_ = 1.964; Control: *t*(4) = -2.946, *p* = 0.042, d_Cohen_ = 1.098], and the SD2 stage [Neo-PRh: *t*(5) = -3.310, *p* = 0.021, d_Cohen_ = 1.372; Control: *t*(4) = -3.121, *p* = 0.035, d_Cohen_ = 1.37].

### Correlation With Lesion Extent

The extent of PRh damage was not significantly correlated with the number of errors on any stage [SD1: *r* = -0.804, *p* = 0.054; SD2: *r* = 0.335; *p* = 0.516; SR: *r* = 0.448, *p* = 0.373; CD: *r* = 0.670, *p* = 0.145; CR: *r* = 0.759, *p* = 0.080; IDS: *r* = 0.249, *p* = 0.634; IDR: *r* = 0.756, *p* = 0.082; EDS: *r* = 0.697, *p* = 0.124]. Similarly, the extent of unintended entorhinal cortex damage was not significantly correlated with the number of errors on any stage [SD1: *r* = -0.021, *p* = 0.968; SD2: *r* = 0.469; *p* = 0.349; SR: *r* = 0.246, *p* = 0.639; CD: *r* = 0.433, *p* = 0.392; CR: *r* = -0.063, *p* = 0.905; IDS: *r* = 0.418, *p* = 0.409; IDR: *r* = -0.105, *p* = 0.843; EDS: *r* = 0.356, *p* = 0.489]. However, it must be acknowledged that the lesions in the 6 Neo-PRh monkeys were similar in extent and had limited variability, ranging only between 70 and 85% (see Table 1). This lack of variability limits the ability to interpret the correlations between lesion extent and task performance.

## Discussion

This is the first study to date that has investigated the impact of neonatal PRh lesions on cognitive flexibility and behavioral inhibition using the ID-ED set-shifting task. The results indicated that Neo-PRh lesions had little impact on the ability of adult monkeys to acquire novel visual discriminations in the SD, CD, and IDS stages, or to complete the reversal stages, but significantly impaired performance on the EDS stage. These results revealed that mechanisms important for visual discrimination learning and behavioral inhibition functioned in the normal range following the early lesions, whereas mechanisms mediating cognitive flexibility were significantly impaired. These findings are discussed in turn.

### Visual Discrimination Learning

Visual discrimination learning involves the formation of stimulus-response associations. In the SD, CD, and IDS stages, monkeys learned which of two stimuli to respond to in order to obtain a reward, and which one to avoid. Monkeys with Neo-PRh lesions completed the visual discrimination stages of the ID/ED task as quickly and accurately as controls. These data confirmed similar findings from the same animals when tested on the 60-pair concurrent discrimination task (personal communication, J. Bachevalier), and indicated that Neo-PRh lesions do not impair simple discrimination learning. Monkeys with PRh lesions incurred in adulthood are also able to perform similar discrimination tasks normally (Gaffan and Murray, 1992; Thornton et al., 1997; Hampton and Murray, 2002). Taken together, these data indicate that the PRh does not play a significant role in stimulus-response association learning.

### Behavioral Inhibition

In the Reversal stages, monkeys learned to switch their response strategies, that is avoid the stimulus previously rewarded and select the previously unrewarded stimulus. This kind of learning involves inhibition of previously acquired stimulus-reward associations. In the current study, monkeys with Neo-PRh lesions were unimpaired on all reversal stages of the ID-ED task. This finding corroborates data from an earlier study with the same Neo-PRh animals in which they were unimpaired in learning 5 concurrent object discrimination reversal problems (personal communication, J. Bachevalier). Previous research has already indicated that the OFC is important to support behavioral inhibition during reversal learning (Dias et al., 1997; Kazama and Bachevalier, 2009; Rygula et al., 2010; Bachevalier et al., 2011). Given that the PRh has robust connections with the OFC, it is surprising that early removal of the inputs from PRh to the OFC did not impact the functioning of the OFC. Although there exist no data on the morphological effects of neonatal PRh lesions on brain reorganization, and more importantly on the maturation of the PFC, one possibility is that the relatively earlier maturation of the OFC in relation to other frontal regions may render this region less impacted by the lack of inputs from the PRh. This resilience could explain the apparent sparing-of-function observed in our Neo-PRh monkeys when performing the reversal stages.

It is also noteworthy that the spared performance of our Neo-PRh group contrasts with the impaired performance of monkeys with adult-onset PRh lesions on similar reversal tasks (Murray et al., 1998; Hampton and Murray, 2002). These different outcomes following neonatal and adult-onset PRh lesions could be related to two important procedural differences between the studies. First, the lesions in the adult monkeys tested by Murray et al. (1998) were created by surgical aspiration, whereas the lesions in our Neo-PRh monkeys were created by injection of neurotoxin. An important difference between these two methods is that the neurotoxin injection destroys only the neurons it contacts, whereas the surgical aspiration destroy fibers within and adjacent to the PRh. Studies directly comparing the impact of these two lesion techniques in other MTL areas indicated that more severe deficits followed aspiration lesions than neurotoxic lesions (Meunier et al., 1999; Glenn et al., 2005). Second, the adult PRh lesions encompassed the entire PRh and large portions of the entorhinal cortex, whereas the neonatal PRh lesions did not. Therefore, damage to other MTL areas, rather than or in addition to the PRh, could also contribute to the more severe reversal learning impairments in monkeys with adult-onset PRh lesions (see Zola-Morgan et al., 1994). Although data from the current study suggest that the PRh does not play a role in behavioral inhibition, comparisons with the adult data highlight a need for future studies to clarify the role of the PRh in reversal learning and behavioral inhibition when lesions are made in adulthood.

### Cognitive Flexibility

Cognitive flexibility involves the ability to switch attention to different sources of information, especially when behavioral responses become unsatisfactory or inadequate. The EDS stage requires flexibility to ignore the previously attended-to dimension of the stimuli (shape) and shift attention to the previously ignored dimension of the stimuli (line). Neo-PRh monkeys had significant difficulty shifting their response strategies during the EDS stage, as indicated by their high error rates. Compared with the normal performance on reversal learning (behavioral inhibition) reported above, these data suggest that Neo-PRh lesions impaired mechanisms of cognitive flexibility.

Given the critical anatomical connections of the PRh with the lateral prefrontal cortex, it is possible that the deficits resulted from direct damage to the PRh or from downstream effects of the neonatal PRh lesions on the normal maturation of other neural structures, especially those with protracted anatomical and functional development, such as the PFC (Fuster, 2002; Overman, 2004; Conklin et al., 2007; Kolb et al., 2010; Perlman et al., 2015). Developmental studies in rodents (Tseng et al., 2009) and monkeys (Bertolino et al., 1997; Chlan-Fourney et al., 2000; Meng et al., 2013) have already demonstrated significant morphological and neurochemical changes in the lateral PFC as a result of early damage to the MTL structures. Given that, as compared to the OFC, the lateral PFC is critical for cognitive flexibility (Dias et al., 1997), the loss of cognitive flexibility after the neonatal PRh lesions may have resulted from maldevelopment of the lateral PFC following early disruption of inputs it receives from the PRh rather than damage to PRh *per se*. If so, one possibility could be that mechanisms of neural plasticity in anterior regions of the PFC that are known to be important to learn WM tasks (as in Riley et al., 2018) may have been altered during development. One way of disentangling these alternative interpretations will require the replication of the current experiments in a group of monkeys that will have received the same PRh lesions in adulthood, i.e., when the PRh lesions will occur at a time when the PFC is fully mature.

### Relationship to Perseverative Responses

A previous report indicated that the performance of the same Neo-PRh monkeys on WM tasks that generated proactive interference was characterized by greater tendencies for perseverative errors, yet the same animals performed normally on a WM task that was devoid of interference (Weiss et al., 2016). This finding suggested that the early lesions did not impact WM processes *per se* but rather altered executive cognitive processes other than WM. In the current study, Neo-PRh monkeys had significant difficulty learning to shift their attention to new perceptual features in the EDS stage, but were able to complete visual discrimination and reversal stages as quickly and accurately as controls. These findings provide a potential explanation for the increase in perseverative errors in the WM tasks reported earlier (Weiss et al., 2016) by implying that deficient cognitive flexibility is the likely source of the increased perseverative errors.

The critical involvement of the dorsolateral PFC is well established in WM processes of monitoring/manipulation (Goldman and Rosvold, 1970; Alexander and Goldman, 1978; Petrides and Milner, 1982; Petrides, 1991a,b, 1995, 2000; Miller and Cohen, 2001), whereas ventrolateral and medial PFC regions are involved with perseveration and cognitive flexibility (Rogers et al., 2000; Monchi et al., 2001; Burnham et al., 2010; Bissonette et al., 2013). It is therefore noteworthy that the PFC regions critical for cognitive flexibility receive comparably heavier perirhinal inputs than do the regions involved in WM (dorsolateral PFC), and comparably fewer from other areas like inferotemporal cortex, parahippocampal cortex, and the hippocampus (Suzuki and Amaral, 1994a,b; Cavada et al., 2000; Lavenex et al., 2002; Croxson et al., 2005; Kondo et al., 2005; Saunders et al., 2005). This distinct pattern of PRh-PFC anatomical connectivity could explain why removal of the PRh had a more profound impact on mechanisms relying on the ventrolateral PFC (cognitive flexibility) than mechanisms relying on dorsolateral PFC (WM).

## Conclusion

Infancy represents a stage of development characterized by increased levels of neural plasticity (for reviews see Kolb and Gibb, 2007, 2011; Takesian and Hensch, 2013). Perturbation of the brain at this early stage of development may lead to increased opportunity for compensation, but may also increase vulnerability to maldevelopment. In the current study, Neo-PRh lesions profoundly impaired set shifting, whereas data on the effects of extended damage to MTL structures (including the PRh) in adulthood indicated that set-shifting abilities were not impacted (Owen et al., 1991). This dissociation suggests that mechanisms of cognitive flexibility were more severely affected by the early damage than after the adult-onset damage. Given the early timing of the neonatal lesions, the deficits in cognitive flexibility may instead represent downstream effects of the neonatal lesions on the normal development and maturation of the brain area important for flexible cognition and preventing perseverative responding, such as the vlPFC (Iversen and Mishkin, 1970; Owen et al., 1991; Dias et al., 1997; Rogers et al., 2000; Monchi et al., 2001; Baxter et al., 2009; Burnham et al., 2010; Bissonette et al., 2013). The protracted anatomical and functional development of this prefrontal area has been well established (Fuster, 2002; Conklin et al., 2007; Kolb and Gibb, 2011), and a number of morphological and neurochemical changes in the lateral PFC have been reported following early damage to other MTL structures (Bertolino et al., 1997; Chlan-Fourney et al., 2000; Tseng et al., 2008; Meng et al., 2013). Taken together, the current findings indicate that early PRh damage may have had a profound impact on the development of flexible cognition and suggest altered functionality of the vlPFC. Future studies will need to assess the effects of Neo-PRh lesions on prefrontal morphology, and to document whether there are windows of increased vulnerability during which early lesions have differential impacts on the development of the PFC.

## Author Contributions

AW, JW, and RR acquired the data. AW and JB designed the experiments, analyzed the data, and prepared the manuscript.

## Conflict of Interest Statement

The authors declare that the research was conducted in the absence of any commercial or financial relationships that could be construed as a potential conflict of interest.
